# A Compact Snake Optimization Algorithm in the Application of WKNN Fingerprint Localization

**DOI:** 10.3390/s23146282

**Published:** 2023-07-10

**Authors:** Weimin Zheng, Senyuan Pang, Ning Liu, Qingwei Chai, Lindong Xu

**Affiliations:** College of Computer Science and Engineering, Shandong University of Science and Technology, Qingdao 266590, China; zhengweimin@sdust.edu.cn (W.Z.); sdust_psy@163.com (S.P.); sdust_liuning@163.com (N.L.); xulindong777@163.com (L.X.)

**Keywords:** compact, snake optimization, RSSI localization, WKNN

## Abstract

Indoor localization has broad application prospects, but accurately obtaining the location of test points (TPs) in narrow indoor spaces is a challenge. The weighted K-nearest neighbor algorithm (WKNN) is a powerful localization algorithm that can improve the localization accuracy of TPs. In recent years, with the rapid development of metaheuristic algorithms, it has shown efficiency in solving complex optimization problems. The main research purpose of this article is to study how to use metaheuristic algorithms to improve indoor positioning accuracy and verify the effectiveness of heuristic algorithms in indoor positioning. This paper presents a new algorithm called compact snake optimization (cSO). The novel algorithm introduces a compact strategy to the snake optimization (SO) algorithm, which ensures optimal performance in situations with limited computing and memory resources. The performance of cSO is evaluated on 28 test functions of CEC2013 and compared with several intelligent computing algorithms. The results demonstrate that cSO outperforms these algorithms. Furthermore, we combine the cSO algorithm with WKNN fingerprint positioning and RSSI positioning. The simulation experiments demonstrate that the cSO algorithm can effectively reduce positioning errors.

## 1. Introduction

Metaheuristic algorithms are computational algorithms that draw inspiration from the behavioral patterns of various natural creatures. Particle swarm optimization (PSO) is a classical metaheuristic algorithm proposed by Kennedy and Eberhart based on birds’ feeding behavior. It involves a global optimal position and an individual optimal position. In each iteration, these two locations are updated according to the fitness values to obtain the optimal solution to the problem. The PSO algorithm’s attributes, including ease of implementation, fast optimization, and limited parameters, enable its application to numerous fields [1,2,3]. Due to the excellent performance and broad application of PSO, many research endeavors have dedicated substantial efforts to this domain. Zhan et al. proposed adaptive PSO (APSO) by using a parameter adaptation scheme and an elitist learning strategy [4]. Neri et al. proposed compact PSO (cPSO) to reduce memory usage [5]. Zheng et al. proposed compact adaptive PSO (cAPSO) by combining the compact strategy with APSO for mobile sensor localization [6]. Liang et al. proposed comprehensive learning PSO (CLPSO), which uses a novel learning strategy to enable the diversity of the swarm and discourage premature convergence [7]. Hu et al. proposed a multisurrogate assisted binary PSO, which develops a modified strategy for updating the population in binary PSO [8]. Inspired by the hunting behavior of whales, Mirjalili et al. proposed the whale optimization algorithm (WOA), which divides whaling into two phases: surround prey and bubblenet attacking method [9]. The artificial bee colony (ABC) algorithm eliminates the influence of the global best individual by allowing bees to become onlooker bees or employed bees. Employed bees explore the environment around their position, and onlooker bees select an employed bee to follow based on its fitness value, ultimately concluding the algorithm by returning the best fitness value discovered by all bees [10]. The black hole algorithm (BH) was devised by Hatamlou, drawing inspiration from the phenomenon of black holes [11]. Zheng et al. proposed an opposition-based learning black hole (OBH) algorithm to improve the accuracy of mobile wireless sensor network localization [12]. Zheng et al. proposed a new black hole algorithm by developing a compact strategy and an elitist learning strategy to improve the ability to jump out of the local optimum [13]. Zheng et al. also proposed the Levy Flight Edge Regeneration Black Hole algorithm (LEBH) to speed up convergence and enhance optimization capabilities [14]. Furthermore, Pan et al. conducted a comprehensively state-of-the-art investigation of the engineering applications employed by binary metaheuristic algorithms [15].

Wireless sensor networks (WSNs) have been widely used in various fields, such as safety monitoring of underground mines [16], home automation [17], and agriculture [18]. The localization of sensor nodes plays a significant role in such applications of WSNs. WSNs are also used for forest fire detection [19] and environmental monitoring [20]. To obtain accurate information about monitored objects, the positioning and status of sensors are critical for WSNs. The current location service field can be divided into outdoor and indoor. Most outdoor positioning is based on satellite positioning, such as global positioning systems (GPS), the principle of which is to calculate the user’s position information using the relationship between the receiver and the satellite. However, building occlusion weakens satellite signals in indoor environments, leading to inaccurate positioning results [21]. Therefore, accurate indoor localization has become a research frontier in the localization field, offering great potential for research institutions. There are two main categories of WSN-based indoor localization techniques: range-based (arrival-based) and fingerprint-based [22]. In the range-based or arrival-based field, many researchers have introduced several techniques, such as angle of arrival (AOA) [23], time difference of arrival (TDOA) [24], and received signal strength indicator (RSSI) [25,26].

Traditional fingerprint-based localization usually uses RSSI to measure the distance between the transmitter and the receiver and then match the location [27]. However, the accuracy of the matching process is often impacted by various factors, including multipath effects, shadow effects, and non-line-of-sight (NLOS) propagation, which adversely affect localization precision [28]. To improve accuracy, Ma et al. proposed an RSSI ranking fingerprint localization system that considers the ranking of the access point (AP) [29]. Oh et al. proposed an adaptive K-nearest neighbor algorithm in which reference points (RPs) are involved in the positioning process only when their characteristic distances toward the corresponding test point (TP) are under a certain threshold [30].

The SO algorithm has characteristics such as fast convergence speed, strong search capability, robustness, and low algorithm complexity. Wireless sensor nodes have the characteristics of low energy consumption and low memory, requiring low algorithm complexity and a small population size. However, the population size is related to the diversity of the algorithm and therefore affects its performance. In order to reduce the population without affecting algorithm performance, we introduced a compact strategy to the SO algorithm that simulates the population with probability. In this paper, a compact strategy is applied to improve the performance and reduce the memory usage of snake optimization (SO). The compact snake optimization (cSO) algorithm is tested in 28 test functions of CEC2013 and compared with the common heuristic algorithms SO, ABC, fish migration optimization (FMO), grey wolf optimization (GWO), and differential evolution (DE). The simulation results show that cSO achieves better performance. Furthermore, we combine the cSO algorithm with WKNN fingerprint positioning and RSSI positioning. The simulation experiments demonstrate that the cSO algorithm can effectively reduce positioning errors.

The remainder of this paper is organized as follows. Section 2 introduces the SO algorithm, RSSI localization, and the weighted k-nearest neighbor (WKNN) method used in WSN localization. Section 3 provides a detailed explanation of the compact strategy employed in cSO and the combination of cSO and WKNN fingerprint localization. The simulation results are analyzed in Section 4 to discuss the efficiency of the algorithm and its effectiveness when applied to WKNN fingerprint localization. Section 5 provides a summary of the paper.

## 2. Related Work

### 2.1. Snake Optimizer

The SO algorithm is a new intelligent optimization algorithm proposed by Hashim et al. that turns the feeding, fighting, and mating behavior of snakes into a mathematical mode [31,32,33,34]. Feeding is divided into two phases: the exploration phase and the exploitation phase. The survival strategies of snakes are complex and intriguing. Unlike other metaheuristic algorithms, snake optimization divides the population into males and females. SO is initialized using randomly generated populations. In addition, as snakes are cold-blooded animals, the temperature has a decisive influence on their feeding and copulatory behavior.

The temperature (Temp) is calculated using the following equation.

(1) $$Temp=exp(\frac{-t}{T}),$$

where *t* refers to the current iteration, and *T* is the maximum number of iterations.

The quantity of food (*Q*) can be defined by Equation (2).

(2) $$Q=0.5\times exp(\frac{t-T}{T}).$$

Q<0.25 indicates inadequate food in the environment, meaning that snakes are in the exploration phase and search randomly for food.

The exploration phase is expressed as follows:

(3) $$X_{i,m}^{t+1}=X_{random,m}^{t}\pm C_{2}\times A_{m}\times \left(\left(X_{max}-X_{min}\right)\times rand+X_{min}\right),$$

(4) $$X_{i,f}^{t+1}=X_{random,f}^{t}\pm C_{2}\times A_{f}\times \left(\left(X_{max}-X_{min}\right)\times rand+X_{min}\right),$$

where $X_{i,m}^{t+1}$ and $X_{i,f}^{t+1}$ refer to the positions of the *i*-th male and female, respectively, and $X_{random,m}^{t}$ and $X_{random,f}^{t}$ refer to the positions of individuals randomly selected from the male or female population. $C_{2}$ is set to 0.05, rand is a random value ranging from 0 to 1, and *t* is the current iteration of the algorithm. $A_{m}$ and $A_{f}$ indicate the ability of males and females to find food, which can be represented by Equations (5) and (6), respectively.

(5) $$A_{m}=exp\left(\frac{-f_{rand,m}}{f_{i,m}}\right),$$

(6) $$A_{f}=exp\left(\frac{-f_{rand,f}}{f_{i,f}}\right),$$

where $f_{rand,m}$ and $f_{rand,f}$ refer to the fitness of $X_{rand,m}$ and $X_{rand,f}$, respectively, and $f_{i,m}$ and $f_{i,f}$ are the fitness values of the *i*-th individual in the male and female groups, respectively.

In the exploitation phase, there is enough food available in the environment. If the temperature > 0.6, snakes continue to search for food. Male and female positions can be updated according to Equation (7).

(7) $$X_{i,j}^{t+1}=X_{food}^{t}\pm C_{3}\times Temp\times rand\times \left(X_{food}-X_{i,j}^{t}\right),$$

where $X_{i,j}$ is the new position of an individual in both the male and female populations.

If temperature < 0.6, snakes enter a fight-or-mating state.

Fight Mode:

(8) $$X_{i,m}^{t+1}=X_{i,m}^{t}\pm C_{3}\times FM\times rand\times \left(X_{best,f}-X_{i,m}^{t}\right),$$

(9) $$X_{i,f}^{t+1}=X_{i,f}^{t+1}\pm C_{3}\times FF\times rand\times \left(X_{best,m}-X_{i,f}^{t+1}\right),$$

(10) $$FM=exp(\frac{-f_{best,f}}{f_{i}}),$$

(11) $$FF=exp(\frac{-f_{best,m}}{f_{i}}),$$

where FM and FF represent males’ and females’ fighting abilities, respectively, and $X_{best,m}$ and $X_{best,f}$ are the positions of the best male and female individuals, respectively. $f_{best,f}$ and $f_{best,m}$ indicate the fitness of $X_{best,f}$ and $X_{best,m}$, respectively.

Mating Mode:

(12) $$X_{i,m}^{t+1}=X_{i,m}^{t}\pm C_{3}\times M_{m}\times rand\times \left(Q\times X_{i,f}^{t}-X_{i,m}^{t}\right),$$

(13) $$X_{i,f}^{t+1}=X_{i,f}^{t}\pm C_{3}\times M_{f}\times rand\times \left(Q\times X_{i,m}^{t}-X_{i,f}^{t}\right),$$

(14) $$MM=exp(\frac{-f_{i,f}}{f_{i,m}}),$$

(15) $$MF=exp(\frac{-f_{i,m}}{f_{i,f}}),$$

where MM and MF represent males’ and females’ mating abilities, respectively.

### 2.2. RSSI Localization

RSSI is a widely used parameter for indoor localization due to its ability to provide information about the strength of the signal between the transmitter and the receiver [35]. It relies on the signal power measurement from an AP to a device. As radio waves attenuate following the inverse-square law, the distance between the AP and the client device can be estimated by analyzing the relationship between the transmitted and received signal strengths. As the number of available APs increases, it is possible to collect more information.

In recent years, many improved methods based on RSSI localization have been proposed. Jondhale et al. proposed a range-free algorithm based on RSSI measurements, namely support vector regression [36]. The support-vector-regression-based localization scheme estimates target locations directly through field measurements, bypassing the need for distance computations. Shin et al. proposed a crossing assistance system based on RSSI measurement and Bluetooth for outdoor and indoor location tracking [37]. Herein, we use the KNN method and support vector machine to overcome the problems of the system to enable accurate outdoor positioning.

For RSSI distance measurement, the receiver determines the distance from the transmitter by measuring the energy of the radio frequency signal. Using the distance information, the location of TP can be obtained by the methods of trilateration, least squares, etc. When a wireless signal is transmitted in the atmosphere, the signal strength decreases with distance due to various factors. The mathematical relationship between signal strength change and propagation distance is expressed as follows:

(16) $$RSSI=A+10\times n\times lg\frac{d}{d_{0}}+N_{0},$$

where RSSI indicates the signal strength at a distance of *d* from the sending node; *A* is the signal strength at a distance of $d_{0}$ from the sending node, which is generally derived from experience or hardware specification definitions; *d* is the distance between nodes; *n* is the signal attenuation index; $d_{0}$ is the reference distance, usually sets to 1 m; and $N_{0}$ is a Gaussian random noise variable.

We use $RSSI^{i}$ to represent the RSSI between TP and the *i*-th AP. The distance ($d_{i}$) between TP and the *i*-th AP is shown in Equation (17).

(17) $$d_{i}=10^{\frac{RSSI^{i}-A}{10\times n}}.$$

### 2.3. Fingerprint Localization

Fingerprint localization is an important indoor localization technology, with many scholars conducting research in this area. Hoang et al. proposed recurrent neural networks for WiFi fingerprinting indoor localization [38]. Ahmed Shokry et al. proposed a quantum fingerprint-based localization algorithm to enable large-scale location tracking systems [39]. Fingerprint localization involves two stages: offline training and online positioning. Before these two stages, depending on the size of the target area, the distribution density of RPs is selected appropriately. The location of each RP is determined based on density and the size of the target area. In the offline training stage, the receiver collects the RSSI from different APs at each arranged RP. Then, the collected data are stored in a database to construct and calibrate the radio map. During the online positioning stage, we use the nearest neighbor (NN), k-nearest neighbor (KNN), and WKNN methods to locate the TP. The introduction of WKNN is as follows. The collected RSSI between the *i*-th RP and *M* APs can be represented as

(18) $$RSSI_{i}=\left(RSSI_{i1},\ldots ,RSSI_{iM}\right),i=1,\ldots ,N.$$

The characteristic distance between the TP and the *i*-th RP is as follows:

(19) $$D_{i}={\left(\sum_{j=1}^{M}{\left(RSSI_{tj}-RSSI_{rij}\right)}^{r}\right)}^{\frac{1}{r}},$$

where $RSSI_{tj}$ represents the RSSI between TP and the *j*-th AP, and $RSSI_{rij}$ represents the RSSI between the *i*-th RP and the *j*-th AP. The calculation result corresponds to the Manhattan distance (MD) and Euclidean distance (ED) when r = 1 and r = 2, respectively. Distances from all RPs to TP are calculated and ranked, and the closest *K* RPs are selected. We use $p_{1}=\left(x_{1},y_{1}\right)$, $p_{2}=\left(x_{2},y_{2}\right)$,…, $p_{k}=\left(x_{k},y_{k}\right)$ to represent the coordinates of RPs. The estimated location of TP $\left(x_{t},y_{t}\right)$ is calculated by the following equation.

(20) $$\begin{array}{c} x_{t}=\sum_{i=1}^{k}\omega_{i}x_{i},\\ y_{t}=\sum_{i=1}^{k}\omega_{i}y_{i}, \end{array}$$

where $\omega_{i}$ represents the weight of $p_{i}$, as shown in Equation (21).

(21) $$\omega_{i}=\frac{D_{i}}{\sum_{j=1}^{k}D_{j}},i=1,2\ldots ,k,$$

where $D_{i}$ represents the distance between the TP and the *i*-th RP.

## 3. Application of cSO for Partition WKNN Fingerprint Localization

This section mainly introduces the compact strategy and its application to the snake optimizer algorithm. Then, we apply the cSO algorithm to indoor positioning algorithms to verify its performance and the improvement of the cSO algorithm in terms of positioning accuracy.

### 3.1. Compact Strategy

The main objective of the compact strategy is to minimize memory usage while maintaining or even enhancing the performance of the original algorithm. Naturally, reducing memory usage leads to improved running speed. In order to reduce memory usage and improve running speed when running the SO algorithm, we introduce the compact strategy into the SO algorithm. Populationless is the most prominent feature of compact algorithms. A virtual population is used instead of the actual population. The virtual population is a probability model that represents the overall movement state of the population. The perturbation vector is defined as $PV^{t}=\left(\mu^{t},\sigma^{t}\right)$, where *t* is the current iteration. μ and σ are the mean and standard deviation of the probability distribution function (PDF), respectively. The PDF and cumulative distribution function (CDF) are computed by Equations (22) and (23), respectively.

(22) $$PDF=\frac{e^{\frac{-{\left(x-\mu \right)}^{2}}{2\sigma^{2}}}\times \sqrt{\frac{2}{\pi }}}{\sigma \times (erf\left(\frac{\mu +1}{\sqrt{2\sigma }}\right)-erf\left(\frac{\mu -1}{\sqrt{2\sigma }}\right))}.$$

(23) $$CDF=\int_{-\infty }^{x}\frac{e^{\frac{-{\left(x-\mu \right)}^{2}}{2\sigma^{2}}}\times \sqrt{\frac{2}{\pi }}}{\sigma \times (erf\left(\frac{\mu +1}{\sqrt{2\sigma }}\right)-erf\left(\frac{\mu -1}{\sqrt{2\sigma }}\right))}dx.$$

The iterative update of the perturbation vector is based on comparison to identify the winner and loser. The following equations are used to update μ and σ.

(24) $$\mu^{t+1}=\mu^{t}+\frac{1}{N_{p}}\left(winner-loser\right),$$

(25) $$\sigma^{t+1}=\sqrt{{\left(\sigma^{t}\right)}^{2}+{\left(\mu^{t}\right)}^{2}+\frac{2}{N_{p}}\left(winner^{2}-loser^{2}\right)},$$

where $N_{p}$ is the number of virtual populations, and *t* represents the current iteration.

The pseudocode of the cSO is shown in Algorithm 1. $X_{mc}^{t}$ and $X_{fc}^{t}$ represent the *t*-th male and female individual generated from PV, respectively. $X_{newm}^{t}$ and $X_{newf}^{t}$ are the *t*-th male and female individual generated by the updating formula, respectively. $X_{m}^{t}$ and $X_{f}^{t}$ are the *t*-th male and female individual of the population, respectively.
**Algorithm** **1.** cSO pseudocode1:Initialize Problem Setting (Dim, UB, LB, Curr_iter t=0, Max_iter *T*)  Initialize $X_{m}$, $X_{f}$ randomly2:**while** (t≤T) **do**  $X_{mc}^{t}$ = generateFrom (PV)  Calculate the fitness value of $X_{mc}$  $X_{fc}^{t}$ = generateFrom (PV)  Calculate the fitness value of $X_{mc}$  Define Temp using Equation (1)  Define Q using Equation (2)3:    **if** (Q<0.25) **then**  Execution Exploration Phase using Equations (3) and (4)4:    **else if** (Q>0.6) **then**  Execution Exploration Phase using Equation (7)5:    **else**6:         **if** (rand>0.6) **then**  Perform fight mode using Equations (8) and (9)7:         **else**  Perform mating mode using Equations (12) and (13)8:         **end if**9:    **end if**  [winner, loser] = compare (fitness $\left(X_{mc}^{t}\right)$, fitness($X_{newm}^{t}$))  $X_{m}^{t}$ = winner  Update the PV disturbance vector by Equations (24) and (25)  [winner, loser] = compare (fitness $\left(X_{fc}^{t}\right)$, fitness($X_{newf}^{t}$))  $X_{f}^{t}$ = winner  Update the PV disturbance vector by Equations (24) and (25)10:**end while**

### 3.2. Partition Method

In order to reduce positioning errors, a partition positioning method is proposed in this paper. Dividing indoor space into corresponding areas according to different rooms. Place RPs at an appropriate density based on area. Unlike ordinary fingerprint localization, partitioned fingerprint localization first determines the partition based on the closest RP to TP, and then selects the K RPs closest to TP from the selected partition for calculation. The partitioning method can eliminate the interference of RPs outside the region and limit the RPs used to calculate the final coordinates to the partition where TP is located, thereby effectively reducing the error of fingerprint localization.

### 3.3. A Combination of the cSO Algorithm with RSSI Localization and Partition WKNN Fingerprint Localization

In the first phase of localization, this paper combines cSO with RSSI localization and uses cSO to discover the coordinate of TP and reduce the localization error. To improve positioning accuracy, we use the following fitness function.

(26) $$fitness=\sqrt{\frac{\sum_{i=1}^{4}{\left(d_{RA}\left(i\right)-d_{TA}\left(i\right)\right)}^{2}}{4}},$$

where $d_{RA}$ represents the distance between the calculated result and APs, and $d_{TA}$ is the distance between TP and APs.

In order to measure the accuracy of localization results, we use Equation (27) to calculate the error.

(27) $$error=\sqrt{{\left(x-x_{t}\right)}^{2}+{\left(y-y_{t}\right)}^{2}},$$

where (x,y) represents the coordinates of computed results, and $\left(x_{t},y_{t}\right)$ represents the actual coordinates of TP.

In the second phase of localization, another TP coordinate is obtained via partition WKNN fingerprint localization.

In the final phase of positioning, the coordinates obtained from the first two stages are weighted and summed to obtain the final coordinates; the formula is shown in Equation (28).

(28) $$\left(x,y\right)=C_{1}\times \left(x_{r},y_{r}\right)+C_{2}\times \left(x_{p},y_{p}\right),$$

where $C_{1}$ and $C_{2}$ are both set to 0.5. The coordinates $\left(x_{r},y_{r}\right)$ and $\left(x_{p},y_{p}\right)$ represent the results computed by cSO-based RSSI positioning and partition WKNN fingerprint localization, respectively.

## 4. Results and Discussion

In this section, we deploy simulation experiments of the algorithm and the new positioning method. The simulation results of the cSO are tested under 28 test functions of CEC2013 [40]. These functions are divided into three main types: unimodal functions, basic multimodal functions, and composition functions. They can evaluate the performance differences between the new algorithm and other algorithms. In addition, we deploy simulation experiments for WSN localization. The simulation outcomes of cSO-based localization indicate that the cSO algorithm can significantly decreases the positioning error.

### 4.1. Simulation Results on CEC2013

In this section, cSO is compared with SO, GWO, FMO, DE, and ABC to evaluate the its performance. Additionally, the performance of each algorithm is compared with that of the cSO algorithm using the Wilcoxon signed rank test with a significance level of α = 0.05. Table 1 displays a comparison of the performance of cSO and that of typical heuristic algorithms. The algorithms used for comparison have a maximum number of iterations of 1000, a population of 30, and dimensions of 30. The maximum number of iterations for cSO is 15,000, the population size is 2, and the dimension is set to 30. The search range requirement in CEC2013 is [−100, 100]. Table 1 presents a performance comparison of cSO and common heuristic algorithms. The symbol “>” indicates that cSO outperforms the other heuristic algorithm, the “=” symbol indicates that the performance of cSO is comparable to that of the other heuristic algorithm, and the “<” symbol indicates that the performance of cSO is inferior to that of the other heuristic algorithm. The final row of Table 1 summarizes the comparison results across all test functions. Table 1 shows that the test performance of cSO is better than that of SO in 17 functions and the same as that of SO in 1 function. The test performance of cSO is better than that of ABC in all functions. The test performance of cSO is better than that of FMO in 25 functions and worse than that of FMO in 3 functions. The test performance of cSO is better than that of GWO in 21 functions, the same as that of GWO in 1 function, and worse than that of DE in 6 functions. Compared with DE, the test performance of cSO is better than that of DE in 16 functions, the same as that of DE in 2 functions, and worse than that of DE in 10 functions. Therefore, the combination of the SO algorithm with the compact strategy results in significantly improved performance compared to common heuristic algorithms.

**Figure 1:**
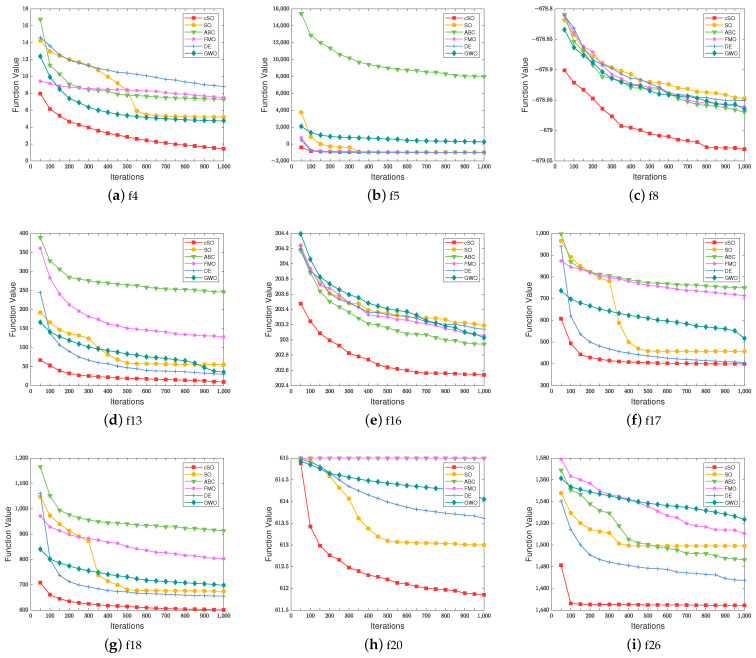
Performance comparison between cSO and common heuristic algorithms.

To further illustrate the algorithm’s effectiveness, we employ convergence curves for evaluation. However, as the convergence of some algorithms is quite similar, the performance differences are not obvious in the convergence curves. Therefore, we choose several representative scattered curves for display. Figure 1 indicates the convergence process of the algorithm on several test functions. The X-axis represents the number of iterations, while the Y-axis represents the fitness values of the different algorithms. A lower fitness value indicates a better performance on the respective test function. Figure 1 shows that the proposed cSO algorithm outperforms other heuristic algorithms on test functions f4, f5, f7, f8, f13, f16, f17, f18, f20, and f26. In the unimodal functions of CEC2013, cSO achieves the optimal value on f4 and f5. In the f4 function, the cSO algorithm outperforms the other five algorithms at the beginning of the iteration and maintains a more stable downward trend as the iteration progresses. In the f5 function, except for the poor performance of the ABC algorithm, cSO, SO, FMO, DE, and GWO can all find a good value at the beginning of the iteration and gradually decrease with the iteration. Although Figure 1b shows that cSO is equal to SO, FMO, and DE in the final results, Table 1 shows that cSO still outperforms them in terms of optimal values. In the basic multimodal functions of CEC2013, cSO achieves the lowest results on f8, f13, f16, f17, f18, and f20. On the f8 and 16 functions, the other five algorithms have similar convergence curves and results, and cSO is significantly better than them throughout the entire iteration cycle. On the f13 and 18 functions, ABC and FMO fall into local optima after a brief search at the beginning of the iteration. SO, GWO, and DE continue to search for optima with each iteration, ultimately achieving better results than the first two algorithms. The cSO achieves a good result in the early stages of the algorithm and gradually decreases with each iteration, ultimately obtaining the best value among all algorithms. In the first 300 iterations of the f17 function, ABC, FMO, and SO have similar convergence curves. After 300 iterations, SO produces better results and significantly reduces the function value, while ABC and FMO fall into local optima. The function value of GWO decreases with iterations, showing a smooth convergence curve. The initial value of DE is not good, but a good result is quickly achieved at the beginning of the iteration, and the function value decreases significantly and continues to decrease as the iteration progresses. The cSO achieves a good result at the beginning and continues to decline for the first 200 iterations, then tends to stagnate. At the beginning of function f20, all algorithms have an almost identical function value. The function value of cSO rapidly decreases, and the convergence curve becomes smoother as the iteration progresses. SO, DE, and GWO have similar convergence curves in the first 200 iterations. Afterwards, the function values of GWO slowly decrease, while DE has a lower convergence curve. The function value of SO rapidly decreases during the 200th to 500th iterations, then tends to stagnate. In the composition functions of CEC2013, cSO achieves the optimal value on f26. The cSO quickly finds the result closest to the optimal value among all algorithms in the first 100 iterations, then converges. The convergence curve of GWO is gentle, and the final result is the worst. FMO exhibits a steeper convergence curve than GWO and obtains a slightly better value than GWO. SO and ABC continuously achieve better results in the first 400 iterations, with the convergence curve rapidly decreasing, then tending to flatten out. The DE makes significant progress in the first 200 iterations, followed by a slow decline in the curve.

### 4.2. Simulation Results of the New Localization Method

To verify the effectiveness of the cSO algorithm in indoor positioning, we compare the new positioning method with RSSI localization and cSO-based RSSI localization by simulation.

In the simulation experiment, a two-dimensional positioning area with a length of 300 m and a width of 300 m is set. Four APs are set at the four vertices of the experimental area, with coordinates of (0,0), (300,0), (300,300), and (0,300). The simulation experiments are carried out using MATLAB 2022a. In order to avoid obtaining the same distance from AP at two centrally symmetric RPs in the positioning area, the transmission powers of the four APs are set to different values in the simulation experiment.

With the aim of verifying the performance of the cSO algorithm proposed in this paper, the performances of RSSI localization based on SO, ABC, FMO, GWO, and DE are compared. Six sets of coordinates of TP are randomly generated, and we use six algorithms to estimate the position of TP and calculate the error.

Then, three groups of controlled experiments are designed according to the number of partitions, RP density, and noise. Each experiment contains six groups of data. In these three simulation experiments, RPs are evenly deployed in the positioning area, and TPs are randomly placed within the experimental field.

#### 4.2.1. RSSI Localization Experiment

**Figure 2:**
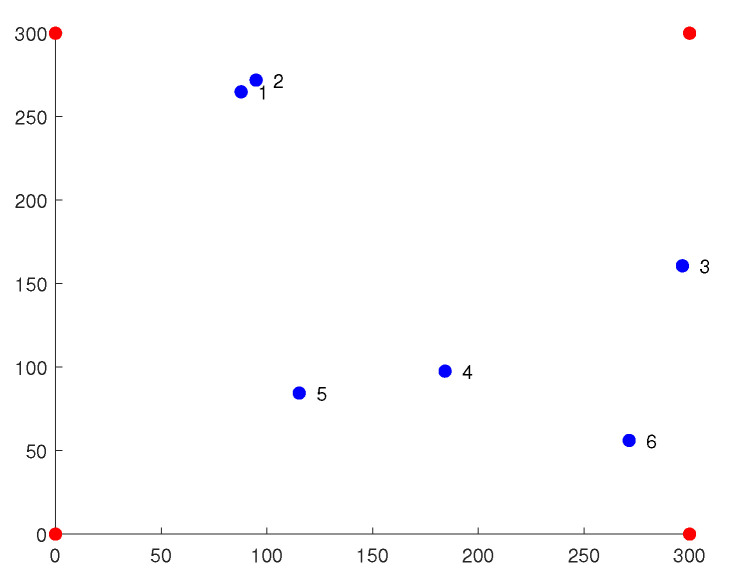
Two-dimensional map of the experimental field.

Simulation experiments are conducted to further validate the reliability of the proposed approach. We randomly place six TPs in the experimental area, as shown in Figure 2. Then, RSSI localization combined with each of the six algorithms is used for calculation, the results of which are shown in Table 2. Based on the experimental data, we can conclude that cSO is superior to other algorithms.

#### 4.2.2. Partition Experiment

**Figure 3:**
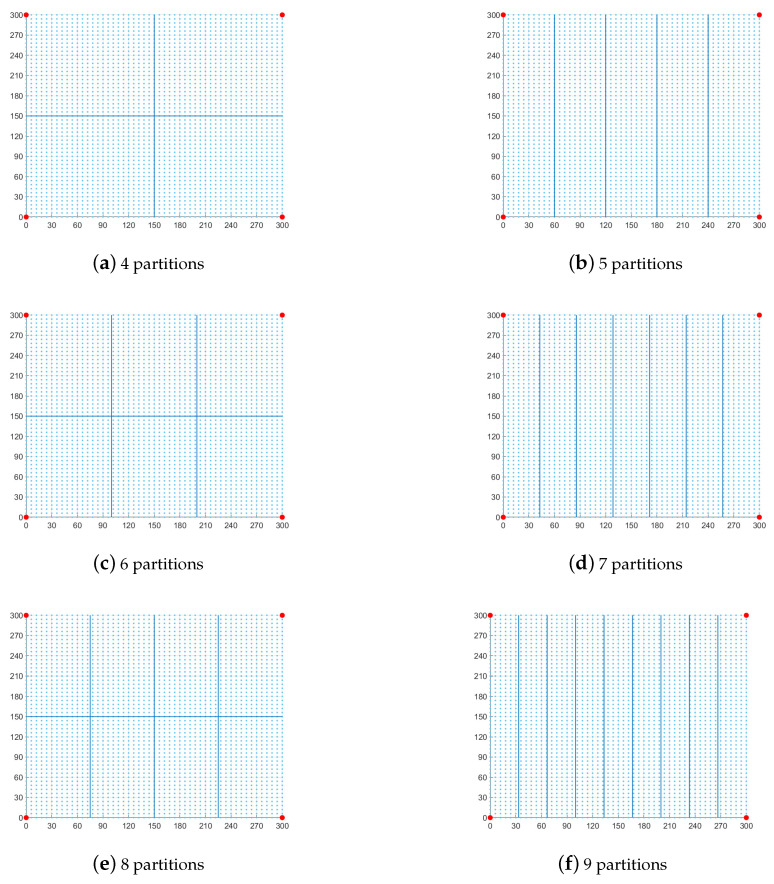
Different partitions of the experimental field.

In the partition experiment, we divide the experimental area into four, five, six, seven, eight, and nine small areas. The different partitions are shown in Figure 3. Table 3 shows the effect of different numbers of partitions on localization error. Table 3 shows that even partitions have better errors than odd partitions in partition WKNN fingerprint localization because even partitions divide the simulation area more evenly. In addition, for a fixed simulation area, too many partitions cause the error to increase. Four-partition WKNN localization is more accurate than RSSI positioning in all partitions. Benefiting from the precise partitioning of WKNN fingerprint localization, the accuracy of the new method is superior to that of cSO-based RSSI localization in all experiments.

#### 4.2.3. Density Experiment

For the purpose of investigating the impact of node density on experimental data, each test has a different density, with RP spacing is set to one, two, three, four, five, and six. Table 4 shows that the best density is 3 m between RPs. Excessive density leads to increased computational complexity and incorrect distance calculation. Low density results in too few RPs, making it difficult to accurately calculate the distance between RPs and TPs.

#### 4.2.4. Noise Experiment

With the intention of verifying the effect of noise on positioning accuracy, six groups with different noise levels (three, four, five, six, seven, and eight) are set up in this experiment. Table 5 shows that as the noise increases, the errors of all localization methods also increase. Noise directly affects the positioning errors of localization methods.

## 5. Conclusions

In this paper, we propose cSO as an improvement of WSN localization, which employs a compact strategy. By conducting experiments on 28 classical test functions, we comprehensively evaluated the proposed algorithm’s performance. The simulation results demonstrate that the novel algorithm achieves competitive performance and good stability. Furthermore, we applied the cSO algorithm to localization and compared its performance with that of WKNN fingerprint localization, RSSI localization, and cSO-based RSSI localization. The results show that the cSO algorithm and fitness function proposed in this article can effectively reduce positioning errors.

In the future, we plan to propose new strategies and explore the potential benefits of combining new algorithms with the WSN localization method. These efforts have the potential to yield excellent results. 

## Figures and Tables

**Table 1:** Performance comparison between cSO and common heuristic algorithms, as well as the Wilcoxon signed rank test of each algorithm at a significance level of α = 0.05.

Function	cSO		SO		ABC		FMO		GWO		DE
$f_{1}$	$-9.38\times 10^{2}$	<	$-1.40\times 10^{3}$	>	$1.11\times 10^{4}$	>	$-5.33\times 10^{2}$	>	$3.39\times 10^{2}$	<	$-1.39\times 10^{3}$
$f_{2}$	$2.06\times 10^{7}$	<	$1.95\times 10^{7}$	>	$3.78\times 10^{8}$	<	$6.75\times 10^{6}$	>	$3.31\times 10^{7}$	>	$8.99\times 10^{7}$
$f_{3}$	$3.84\times 10^{9}$	=	$4.65\times 10^{9}$	>	$1.41\times 10^{17}$	>	$9.32\times 10^{9}$	>	$9.62\times 10^{9}$	<	$3.79\times 10^{9}$
$f_{4}$	$2.78\times 10^{4}$	>	$5.09\times 10^{4}$	>	$7.22\times 10^{4}$	>	$6.70\times 10^{4}$	>	$4.72\times 10^{4}$	>	$8.98\times 10^{4}$
$f_{5}$	$-1.00\times 10^{3}$	>	$-9.74\times 10^{2}$	>	$7.48\times 10^{3}$	>	$-9.86\times 10^{2}$	>	$9.15\times 10^{1}$	>	$-9.95\times 10^{2}$
$f_{6}$	$-7.98\times 10^{2}$	>	$-7.86\times 10^{2}$	>	$1.17\times 10^{3}$	<	$-8.43\times 10^{2}$	>	$-6.98\times 10^{2}$	<	$-8.58\times 10^{2}$
$f_{7}$	$-7.15\times 10^{2}$	>	$-6.60\times 10^{2}$	>	$1.25\times 10^{5}$	>	$-6.01\times 10^{2}$	>	$-7.15\times 10^{2}$	>	$-7.01\times 10^{2}$
$f_{8}$	$-6.79\times 10^{2}$	>	$-6.79\times 10^{2}$	>	$-6.79\times 10^{2}$	>	$-6.79\times 10^{2}$	>	$-6.79\times 10^{2}$	>	$-6.79\times 10^{2}$
$f_{9}$	$-5.70\times 10^{2}$	>	$-5.70\times 10^{2}$	>	$-5.61\times 10^{2}$	>	$-5.59\times 10^{2}$	=	$-5.78\times 10^{2}$	>	$-5.63\times 10^{2}$
$f_{10}$	$-1.58\times 10^{2}$	<	$-4.34\times 10^{2}$	>	$1.69\times 10^{3}$	<	$-3.55\times 10^{2}$	>	$-1.00\times 10^{1}$	<	$-4.32\times 10^{2}$
$f_{11}$	$-3.38\times 10^{2}$	>	$-3.05\times 10^{2}$	>	$6.90\times 10^{1}$	>	$-1.40\times 10^{2}$	>	$-2.72\times 10^{2}$	=	$-3.66\times 10^{2}$
$f_{12}$	$-7.52\times 10^{1}$	<	$-9.59\times 10^{1}$	>	$1.59\times 10^{2}$	>	$2.82\times 10^{1}$	<	$-1.29\times 10^{2}$	=	$-7.42\times 10^{1}$
$f_{13}$	$1.65\times 10^{1}$	>	$5.16\times 10^{1}$	>	$2.48\times 10^{2}$	>	$1.37\times 10^{2}$	>	$3.71\times 10^{1}$	>	$2.63\times 10^{1}$
$f_{14}$	$3.78\times 10^{3}$	<	$1.78\times 10^{3}$	>	$4.76\times 10^{3}$	>	$7.37\times 10^{3}$	>	$3.86\times 10^{3}$	<	$9.23\times 10^{2}$
$f_{15}$	$6.63\times 10^{3}$	>	$7.47\times 10^{3}$	>	$7.69\times 10^{3}$	>	$7.76\times 10^{3}$	<	$5.23\times 10^{3}$	>	$7.93\times 10^{3}$
$f_{16}$	$2.03\times 10^{2}$	>	$2.03\times 10^{2}$	>	$2.03\times 10^{2}$	>	$2.03\times 10^{2}$	>	$2.03\times 10^{2}$	>	$2.03\times 10^{2}$
$f_{17}$	$3.98\times 10^{2}$	>	$4.5\times 10^{2}$	>	$7.37\times 10^{2}$	>	$7.03\times 10^{2}$	>	$5.13\times 10^{2}$	>	$4.06\times 10^{2}$
$f_{18}$	$6.20\times 10^{2}$	>	$6.78\times 10^{2}$	>	$9.08\times 10^{2}$	>	$8.03\times 10^{2}$	>	$6.92\times 10^{2}$	>	$6.52\times 10^{2}$
$f_{19}$	$5.12\times 10^{2}$	<	$5.10\times 10^{2}$	>	$2.66\times 10^{5}$	>	$5.15\times 10^{2}$	>	$1.15\times 10^{3}$	<	$5.10\times 10^{2}$
$f_{20}$	$6.12\times 10^{2}$	>	$6.13\times 10^{2}$	>	$6.15\times 10^{2}$	>	$6.15\times 10^{2}$	>	$6.14\times 10^{2}$	>	$6.14\times 10^{2}$
$f_{21}$	$1.01\times 10^{3}$	<	$9.69\times 10^{2}$	>	$2.72\times 10^{3}$	>	$1.06\times 10^{3}$	>	$1.95\times 10^{3}$	<	$9.92\times 10^{2}$
$f_{22}$	$4.82\times 10^{3}$	<	$2.97\times 10^{3}$	>	$6.05\times 10^{3}$	>	$8.86\times 10^{3}$	>	$4.85\times 10^{3}$	<	$2.89\times 10^{3}$
$f_{23}$	$7.23\times 10^{3}$	>	$8.17\times 10^{3}$	>	$9.36\times 10^{3}$	>	$9.44\times 10^{3}$	<	$5.66\times 10^{3}$	>	$8.96\times 10^{3}$
$f_{24}$	$1.27\times 10^{3}$	>	$1.28\times 10^{3}$	>	$1.30\times 10^{3}$	>	$1.30\times 10^{3}$	<	$1.26\times 10^{3}$	>	$1.29\times 10^{3}$
$f_{25}$	$1.39\times 10^{3}$	<	$1.39\times 10^{3}$	>	$1.42\times 10^{3}$	>	$1.42\times 10^{3}$	<	$1.38\times 10^{3}$	<	$1.39\times 10^{3}$
$f_{26}$	$1.44\times 10^{3}$	>	$1.50\times 10^{3}$	>	$1.48\times 10^{3}$	>	$1.49\times 10^{3}$	>	$1.53\times 10^{3}$	>	$1.47\times 10^{3}$
$f_{27}$	$2.32\times 10^{3}$	<	$2.26\times 10^{3}$	>	$2.62\times 10^{3}$	>	$2.53\times 10^{3}$	<	$2.18\times 10^{3}$	>	$2.51\times 10^{3}$
$f_{28}$	$2.06\times 10^{3}$	>	$2.55\times 10^{3}$	>	$7.23\times 10^{3}$	>	$2.41\times 10^{3}$	>	$2.96\times 10^{3}$	<	$1.88\times 10^{3}$
</=/>	−		10/1/17		0/0/28		3/0/25		6/1/21		10/2/16

**Table 2:** Position error of each algorithm.

Coordinates	cSO	SO	GWO	ABC	FMO	DE
(88,265)	4.743 m	5.564 m	7.045 m	5.122 m	7.128 m	6.173 m
(95,272)	17.040 m	20.676 m	28.224 m	28.905 m	21.632 m	30.540 m
(297,161)	16.162 m	18.501 m	21.414 m	29.230 m	25.908 m	27.455 m
(184,98)	10.663 m	12.734 m	12.104 m	13.702 m	12.880 m	13.986 m
(115,84)	6.635 m	9.897 m	9.558 m	8.146 m	8.753 m	9.798 m
(271,56)	9.71 m	12.344 m	13.175 m	12.426 m	11.441 m	11.930 m

**Table 3:** Position error of each localization method under different partitions.

Partitions	WKNN	RSSI	cSO−RSSI	New Method
4	11.33 m	17.37 m	11.40 m	8.46 m
5	18.58 m	18.91 m	11.91 m	11.94 m
6	15.50 m	17.12 m	11.51 m	9.91 m
7	25.75 m	17.10 m	11.88 m	15.14 m
8	18.63 m	16.42 m	11.62 m	11.84 m
9	31.16 m	18.12 m	12.04 m	17.73 m

**Table 4:** Position error of each localization method under different densities.

Density	WKNN	RSSI	cSO−RSSI	New Method
1	11.29 m	18.76 m	12.94 m	9.53 m
2	12.77 m	17.78 m	12.37 m	9.44 m
3	11.84 m	16.82 m	12.02 m	8.60 m
4	14.04 m	16.85 m	11.00 m	9.42 m
5	16.25 m	11.87 m	11.11 m	8.92 m
6	13.20 m	16.88 m	11.81 m	9.24 m

**Table 5:** Position error of each localization method under different noise levels.

Noise	WKNN	RSSI	cSO−RSSI	New Method
3	10.21 m	12.81 m	9.06 m	6.94 m
4	9.66 m	14.06 m	10.05 m	7.14 m
5	12.46 m	14.60 m	10.70 m	8.48 m
6	13.45 m	17.30 m	12.54 m	8.88 m
7	14.46 m	18.47 m	13.05 m	10.57 m
8	17.58 m	20.39 m	14.33 m	12.57 m

## Data Availability

The data are contained within the article.

## Abbreviations

The following abbreviations are used in this manuscript:

PSO	Particle swarm optimization
APSO	Adaptive particle swarm optimization
cPSO	Compact particle swarm optimization
CLPSO	Comprehensive learning particle swarm optimization
WOA	Whale optimization algorithm
ABC	Artificial bee colony
BH	Black hole
OBH	Opposition-based learning black hole
LEBH	Levy flight edge regeneration black hole
WSN	Wireless sensor network
GPS	Global positioning system
AOA	Angle of arrival
TDOA	Time difference of arrival
RSSI	Received signal strength indicator
NLOS	Non-line of sight
AP	Access point
RP	Reference point
TP	Test point
SO	Snake optimization
cSO	Compact snake optimization
FMO	Fish migration optimization
GWO	Grey wolf optimization
DE	Differential evolution
WKNN	Weighted k-nearest neighbor
NN	Nearest neighbor
KNN	K-nearest neighbor
MD	Manhattan distance
ED	Euclidean distance
PDF	Probability distribution function
CDF	Cumulative distribution function
